# Supplementary material for: Osteoclast-Driven Osteogenesis, Bone Remodeling and Biomaterial Resorption: A New Profile of BMP2-CPC-Induced Alveolar Bone Regeneration
Source: Int J Mol Sci. 2022 Oct 13;23(20):12204. doi: 10.3390/ijms232012204 (PMC9602653; doi:10.3390/ijms232012204)
Supplement: Supplementary file 1 [file ijms-23-12204-s001.zip › ijms-1924395-supplementary.pdf]

Supplementary Materials

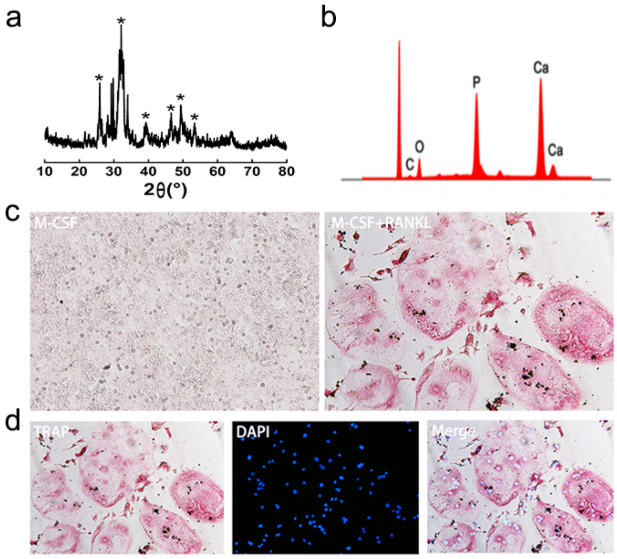

Figure S1. (**a**). XRD patterns of CPC (pentagrams mark the specific peaks of hydroxyapatite); (**b**). EDS of CPC particles; (**c**). Identification of BMM differentiation; (**d**). Osteoclasts double stained with TRAP and DAPI.
